# PTPA Governs Stress-Responsive Differentiation and Metabolic Homeostasis in *Toxoplasma gondii*

**DOI:** 10.3390/cells14110835

**Published:** 2025-06-03

**Authors:** Zhu Ying, Yuntong Wu, Yanqun Pei, Zheng Shang, Jing Liu, Qun Liu

**Affiliations:** 1 National Key Laboratory of Veterinary Public Health and Safety, College of Veterinary Medicine, China Agricultural University, Beijing 100083, China; yzyingzhu@163.com (Z.Y.); wuyuntongmail@163.com (Y.W.); 18349325943@163.com (Y.P.); 18797360881@163.com (Z.S.); 2 Key Laboratory of Animal Epidemiology of the Ministry of Agriculture and Rural Affairs, College of Veterinary Medicine, China Agricultural University, Beijing 100083, China; 3 National Animal Protozoa Laboratory, College of Veterinary Medicine, China Agricultural University, Beijing 100083, China

**Keywords:** *Toxoplasma gondii*, phosphotyrosyl phosphatase activator, bradyzoite differentiation, amylopectin metabolism, LB-100

## Abstract

The protozoan parasite *Toxoplasma gondii* transitions between acute (tachyzoite) and chronic (bradyzoite) stages, enabling lifelong persistence in hosts. Iron depletion triggers bradyzoite differentiation, with the phosphotyrosyl phosphatase activator (PTPA) identified as a key regulator. Here, we define PTPA’s role in *T. gondii* pathogenesis. PTPA forms a ternary complex with PP2A A/C subunits, validated by reciprocal pull-down assays. Depleting PTPA impaired tachyzoite proliferation, invasion, and gliding motility, while stress-induced bradyzoites exhibited defective cyst formation and vacuolar swelling. Metabolic dysregulation included amylopectin accumulation and lipid droplet proliferation. The PP2A inhibitor LB-100 phenocopied PTPA depletion, suppressing tachyzoite growth and bradyzoite differentiation. TgPTPA emerges as a linchpin coordinating PP2A activity, metabolic flux, and lifecycle transitions. Its dual roles in acute virulence and chronic persistence, combined with LB-100’s efficacy, position the PTPA–PP2A axis as a promising target for antitoxoplasmosis strategies.

## 1. Introduction

*Toxoplasma gondii*, an obligate intracellular protozoan parasite, poses a significant global health burden [1], particularly for immunocompromised individuals and pregnant women. Its remarkable adaptability stems from its ability to transition between the rapidly replicating tachyzoite stage, responsible for acute infection, and the dormant bradyzoite stage, which enables lifelong persistence within host tissues [2]. This developmental plasticity ensures immune evasion and complicates therapeutic interventions [3], underscoring the need to elucidate molecular regulators governing its lifecycle. Current in vitro models to study tachyzoite-to-bradyzoite differentiation employ diverse stressors, including alkaline conditions (pH 8.0–8.2) [4], arginine starvation [5], heat shock [4], and chemical treatment such as tunicamycin [6] or atovaquone [7]. Our prior work identified iron depletion as a novel exogenous stressor triggering spontaneous bradyzoite conversion [8], with the phosphotyrosyl phosphatase activator (PTPA) protein emerging as a critical mediator of iron deficiency-induced differentiation [8].

The PTPA is an evolutionarily conserved protein critical for the activation and structural stabilization of protein phosphatase 2A (PP2A), a master regulator of dephosphorylation-dependent signaling across eukaryotes [9]. PP2A, a heterotrimeric serine/threonine phosphatase, governs diverse cellular processes—including cell division, proliferation, motility, and stress adaptation—through its catalytic (C) subunit, scaffold (A) subunit, and variable regulatory subunits [10]. PTPA was initially characterized for its ATP/Mg^2+^-dependent ability to restore the latent phosphotyrosine phosphatase activity of PP2A in vitro [11]. Subsequent studies revealed its dual role as a peptidyl-prolyl cis–trans isomerase targeting Pro190 of the PP2A C subunit [12] and as a molecular chaperone preventing PP2A aggregation [13]. These functions are indispensable for eukaryotic survival, as PTPA knockout in mammalian systems induces cell death and proliferative arrest [14,15], underscoring its centrality to PP2A-dependent signaling fidelity.

In parasitic systems, PTPA homologs exhibit both conserved and lineage-specific functional adaptations. For example, microinjection of recombinant *Plasmodium falciparum* PfPTPA disrupts the G2/M transition in *Xenopus oocytes*, suggesting a conserved role in cell cycle regulation [16]. Conversely, the *Haemonchus contortus* homolog HcPTPA diverges functionally by suppressing host lymphocyte proliferation and inducing apoptosis, highlighting its co-option for immune evasion [17]. Despite these advances, the mechanistic roles of PTPA within apicomplexan parasites remain poorly resolved. In *T. gondii*, the PP2A holoenzyme orchestrates critical processes such as amylopectin metabolism and stage differentiation. Genetic disruption of any PP2A subunit triggers aberrant amylopectin accumulation and blocks tachyzoite-to-bradyzoite conversion [18]. However, the specific contribution of PTPA to PP2A-mediated signaling—particularly its role in stabilizing phosphatase activity or enabling stress adaptation—remains unexplored, creating a critical knowledge gap in understanding how this axis fine-tunes parasite persistence and pathogenesis.

This study elucidates the functional role of *Toxoplasma gondii* phosphotyrosyl phosphatase activator (TgPTPA) in parasite biology. We demonstrate that TgPTPA orchestrates both lytic cycle progression and stress-induced differentiation by modulating PP2A phosphatase activity, positioning it as a master regulator of pathogenesis and a potential therapeutic target.

## 2. Materials and Methods

### 2.1. Parasite Strain, Culture Conditions, and Electroporation

Parasite strain ME49*Δku80Δhxgprt::TIR1* was propagated in human foreskin fibroblasts (HFFs, maintained in our laboratory) cells cultured in Dulbecco’s Modified Eagle’s Medium (DMEM, M&C Gene, Beijing, China) supplemented with 2% fetal bovine serum (FBS, TransGen Biotech, Beijing, China), 100 mg/mL streptomycin, and 100 units/mL penicillin (M&C Gene, Beijing, China). Parasites and cells were maintained in a humidified incubator at 37 °C with 5% CO_2_.

### 2.2. Endogenous Tagging

Tachyzoites were purified by releasing them from host cells via syringe lysis using a 1 mL needle, followed by filtration through a 5 µm polycarbonate membrane to remove cellular debris. For electroporation, CRISPR-Cas9 plasmids targeting the 3′ untranslated region (UTR) downstream of the stop codon of the target gene and an amplicon containing the Ty-AID tag and a drug-selectable marker flanked by 42 bp homology arms were introduced into the parasites. The mixture was subjected to electroporation (Bio-Rad, California, USA), and stable transgenic lines were selected using 3 µM pyrimethamine (Sigma-Aldrich, Missouri, USA). Clonal isolates were obtained through limiting dilution. All primers and plasmids used are listed in the Supplementary Materials [8].

### 2.3. Indirect Immunofluorescence Assay

Cell monolayers grown on coverslips were fixed with 4% paraformaldehyde for 30 min, permeabilized with 0.25% Triton™ X-100 (Solarbio, Beijing, China) for 15–30 min, and blocked with 3% bovine serum (M&C Gene, Beijing, China) at 37 °C for 1 h. Primary antibodies were applied for 1 h at 37 °C, followed by three 5 min PBS washes. Secondary antibodies, including Cy3-conjugated goat anti-rabbit/mouse IgG or FITC-conjugated goat anti-rabbit/mouse IgG (Proteintech, Wuhan, China), were incubated for 1 h at 37 °C. After three additional PBS washes, coverslips were mounted using anti-fade mounting medium containing DAPI (Solarbio, Beijing, China) and imaged using an inverted fluorescence microscope (Olympus, Tokyo, Japan).

### 2.4. Proliferation Assay

Freshly released tachyzoites (1 × 10^5^) were added to HFF monolayers on coverslips. After 2 h of invasion, non-invaded parasites were removed by washing, and infected cells were incubated for 28 h at 37 °C. Cells were processed for indirect immunofluorescence assay (IFA) as described above, using mouse anti-GAP45 polyclonal antibody (laboratory storage) as the primary antibody. The number of parasites per PVs was quantified by fluorescence microscopy for 100 vacuoles. Each experimental group included three technical replicates, and the entire experiment was independently repeated three times.

### 2.5. Invasion Assay

Freshly released tachyzoites (1 × 10^6^) were added to HFF monolayers on coverslips. After 2 h of invasion at 37 °C, the plate was transferred to ice to terminate invasion and washed 5–8 times with ice-cold PBS. Cells were fixed with 4% paraformaldehyde for 10 min, blocked with 5% bovine serum albumin (BSA, M&C Gene, Beijing, China) for 30 min, and incubated with mouse anti-SAG1 (laboratory storage) antibody (30 min, without permeabilization) to label extracellular parasites. Following three PBS washes, cells were permeabilized with 0.25% Triton™ X-100 (15–30 min), re-blocked with 5% BSA, and incubated with rabbit anti-GAP45 antibody (30 min) to label total parasites. Secondary antibodies (Cy3-conjugated goat anti-rabbit IgG and FITC-conjugated goat anti-mouse IgG) were applied for 30 min at 37 °C. Extracellular parasites (FITC-positive) and total parasites (Cy3-positive) were visualized by fluorescence microscopy. The number of invaded parasites was calculated by subtracting extracellular parasites (green) from total parasites (red) per field, normalized to the number of host cell nuclei. Thirty fields per experimental group were analyzed across three technical replicates, with three independent biological repeats [19].

### 2.6. Egress Assay

HFF monolayers were infected with 1 × 10^5^ freshly released tachyzoites. After 2 h of invasion, non-invaded parasites were removed, and infected cells were incubated for 36 h at 37 °C. Egress was triggered by adding medium containing 5% ethanol for 5 min, followed by immediate transfer to ice. Cells were processed for IFA using mouse anti-GAP45 antibody. The egress status of 100 PVs per replicate was assessed by fluorescence microscopy. The experiment included three technical replicates per group, repeated independently three times.

### 2.7. Plaque Assay

HFF monolayers in 12-well plates were infected with 200 freshly released tachyzoites. After 2 h of invasion, non-invaded parasites were removed, and infected cells were cultured in medium for 10–12 days. Cells were fixed with 4% paraformaldehyde (30 min), stained with crystal violet (M&C Gene, Beijing, China), and imaged using a microscope. Plaque areas were quantified using ImageJ 1.52 software. Each experimental group included three technical replicates, with three independent biological repeats.

### 2.8. In Vitro Differentiation Assay

An alkaline induction medium was prepared using RPMI-1640 (Genom-Bio, Hangzhou, China) without sodium bicarbonate buffer, adjusted to pH 8.0–8.2 with 1 M NaOH (Solarbio, Beijing, China), and supplemented with penicillin–streptomycin and 5% FBS. Tachyzoites invading HFF cells were cultured in this alkaline medium in a CO_2_-free incubator for 3–7 days, with daily medium replacement. Differentiation efficiency into bradyzoites was assessed using Dolichos biflorus agglutinin (DBA) staining (Vector Laboratories, California, USA). Following incubation with mouse anti-GAP45 primary antibody, DBA dye was added during secondary antibody incubation. The percentage of DBA-positive vacuoles was quantified by fluorescence microscopy [20].

### 2.9. PTPA Protein 3D Structure Prediction, Phylogenetic Analysis, and Molecular Docking

Amino acid sequences of PTPA from three *Toxoplasma* genotypes were retrieved from the ToxoDB database (https://toxodb.org/toxo/, accessed on 9 October 2024). Three-dimensional structural models of PTPA and its complex with PP2A A/C subunits were predicted using AlphaFold (https://alphafold.ebi.ac.uk/, accessed on 2 November 2024) and visualized with PyMOL 2.2.3 [21]. For phylogenetic analysis, PTPA sequences from other species were obtained from NCBI. A maximum-likelihood phylogenetic tree was constructed using MEGA 10.0.2 and refined with iTOL (https://itol.embl.de/, accessed on 6 November 2024). Molecular docking between the PTPA–PP2A complex and LB-100 was performed using CB-DOCK2 (https://cadd.labshare.cn/cb-dock2/php/index.php, accessed on 9 November 2024), with results visualized in PyMOL 2.2.3 [22,23].

### 2.10. Transmission Electron Microscopy (TEM) of Toxoplasma Ultrastructure

HFF cells in T175 flasks were infected with *Toxoplasma* until PVs dominated the culture. Cells were fixed with 3.5% glutaraldehyde, scraped, pelleted (3000 rpm, 5 min), and post-fixed overnight. Samples were rinsed with 0.1 M phosphate buffer (pH 7.4), fixed with 1% osmium tetroxide for 4 h, and washed again. Dehydration was performed using a graded ethanol series (4 °C, 10 min per step). Samples were infiltrated with 50% epoxy resin in ethanol for 5 h, followed by pure epoxy resin overnight, embedded in molds, and polymerized. Ultrathin sections (70 nm) were cut with an ultramicrotome, stained with uranyl acetate and lead citrate, and imaged by TEM (RuliTEMHT7800; Hitachi, Tokyo, Japan).

### 2.11. Starch Granule Staining and Analysis

HFF monolayers on coverslips were infected with tachyzoites. After treatment, cells were fixed with 4% paraformaldehyde for 10 min, washed three times with PBS, and incubated with periodic acid solution for 5 min at room temperature. Following three PBS washes, samples were stained with Schiff reagent in the dark for 15 min (Solarbio, Beijing, China), washed again, and mounted with anti-fade medium containing DAPI. Periodic acid–Schiff (PAS)-positive parasites were quantified by fluorescence microscopy, assessing both staining positivity and fluorescence intensity.2.12. Lipid Droplet Staining and Analysis

Treated parasites were released from host cells, collected, and washed with PBS. After resuspension in PBS, parasites were incubated with Nile red dye (Solarbio, Beijing, China) for 15 min at 37 °C. Excess dye was removed by centrifugation and PBS washing. Parasites were resuspended in PBS, smeared onto coverslips, fixed, and imaged by fluorescence microscopy to quantify Nile red-stained lipid droplets.

### 2.12. PTPA Protein Expression and Pull-Down

Plasmids encoding TgPTPA (TGME49_283720), PP2A A subunit (TGME49_315670), and PP2A C subunit (TGME49_224220) were constructed using pMAL–His and pMAL–HA backbones. Briefly, backbone sequences were amplified from pMAL–His/HA plasmids, and target genes were amplified from *Toxoplasma* cDNA. Fragments were ligated using an assembly kit and transformed into cloning-competent *E. coli*. Verified plasmids were transformed into expression-competent *E. coli*. Cultures were grown in LB medium with ampicillin at 37 °C, induced with 0.2 mM IPTG for 5 h, and harvested by centrifugation. Bacterial pellets were lysed in buffer (20 mM Tris-HCl, 200 mM NaCl, 1 mM EDTA, 1 mM DTT, pH 7.4) via sonication (3 s on/off cycles, 90 min total). Lysates were clarified by centrifugation, and supernatants were loaded onto MBPSep Dextrin Agarose Resin 6FF (Yeasen Biotechnology, Shanghai, China) columns pre-equilibrated with wash buffer. After washing, proteins were eluted with buffer containing 10 mM maltose (Solarbio, Beijing, China). Purified proteins were verified by SDS-PAGE.

For pulldown assays, magnetic beads (Beyotime, Shanghai, China) were washed with TBS and incubated with tagged proteins for 5 h at 4 °C. Unbound proteins were removed, and beads were incubated with target proteins for 5 h. After washing, bound complexes were eluted, denatured in SDS-PAGE buffer, and analyzed by Western blot.

### 2.13. Proteomic Profiling and Analysis

ME49-iPTPA parasites cultured under normal or alkaline conditions, with or without IAA treatment, were harvested upon partial vacuole rupture. Host cells were scraped, lysed via syringe passage, and filtered through a 45 µm membrane. Parasites were pelleted (2800 rpm, 5 min), washed with PBS, flash-frozen in liquid nitrogen, and stored at −80 °C. Samples were submitted to Shanghai Majorbio Bio-pharm Technology Co., Ltd. (Majorbio, Shanghai, China) for proteomic analysis. Data processing, including volcano plots and GO term enrichment, was performed using the OmicStudio platform (https://www.omicstudio.cn/, accessed on 1 September 2023) [24]. GO and KEGG annotations were sourced from ToxoDB (https://toxodb.org/toxo/, accessed on 1 September 2023).

## 3. Results

### 3.1. Toxoplasma Expresses a Homolog of Phosphotyrosyl Phosphatase Activator Protein

**Figure 1:**
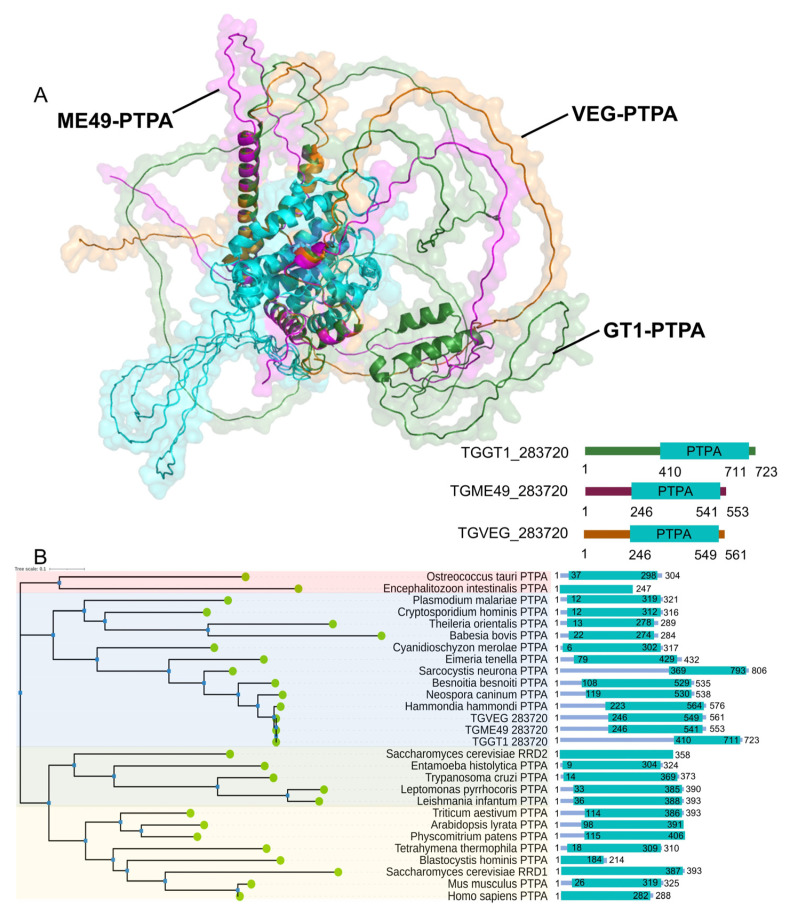
Structural and phylogenetic analysis of PTPA in *Toxoplasma gondii*. (**A**) Predicted structural models of PTPA proteins from *T. gondii* genotypes I (GT1 strain, green), II (ME49 strain, purple), and III (VEG strain, orange), generated using AlphaFold3. The phosphotyrosyl phosphatase activator domain is highlighted in blue. (**B**) Phylogenetic tree of PTPA proteins across eukaryotes, reconstructed using maximum likelihood analysis. *T. gondii* PTPA clusters within a clade closely related to red algae (*Cyanidioschyzon merolae*). Other major branches include green algae (*Ostreococcus taurii*) and yeast homologs (*Saccharomyces cerevisiae* RRD1/RRD2).

Through domain searches and sequence homology alignments, we identified a phosphotyrosine phosphatase activator protein (PTPA) in the *Toxoplasma gondii* database (https://toxodb.org/, accessed on 1 September 2023), which we designated as TgPTPA. Interestingly, the homologous proteins of TgPTPA vary in size among the three *T. gondii* genotypes, Type I (GT1 strain), Type II (ME49 strain), and Type III (VEG strain). However, all of them contain the phosphotyrosyl phosphatase activator domain. Both amino acid sequences and tertiary structures of this domain exhibit high conservation (blue-highlighted regions, Figure 1A). Notably, significant differences are observed in the disordered regions outside the key structural domain. Additionally, we aligned PTPA proteins from a majority of eukaryotes and constructed a phylogenetic tree. These proteins can be broadly divided into three categories: the first group is represented by green algae (*Ostreococcus taauri*), the second group is represented by red algae (*Cyanidioschyzon merolae*), and the third group can be further divided into two sub-branches based on their homology to RRD1 and RRD2 of *Saccharomyces cerevisiaae*. The PTPA proteins of the three *T. gondii* genotypes, like those of nearly all apicomplexan protozoa, belong to the second category, clustering closer to red algae (Figure 1B).

### 3.2. Interaction of PTPA Protein with PP2A A and C Subunits in Toxoplasma gondii

**Figure 2:**
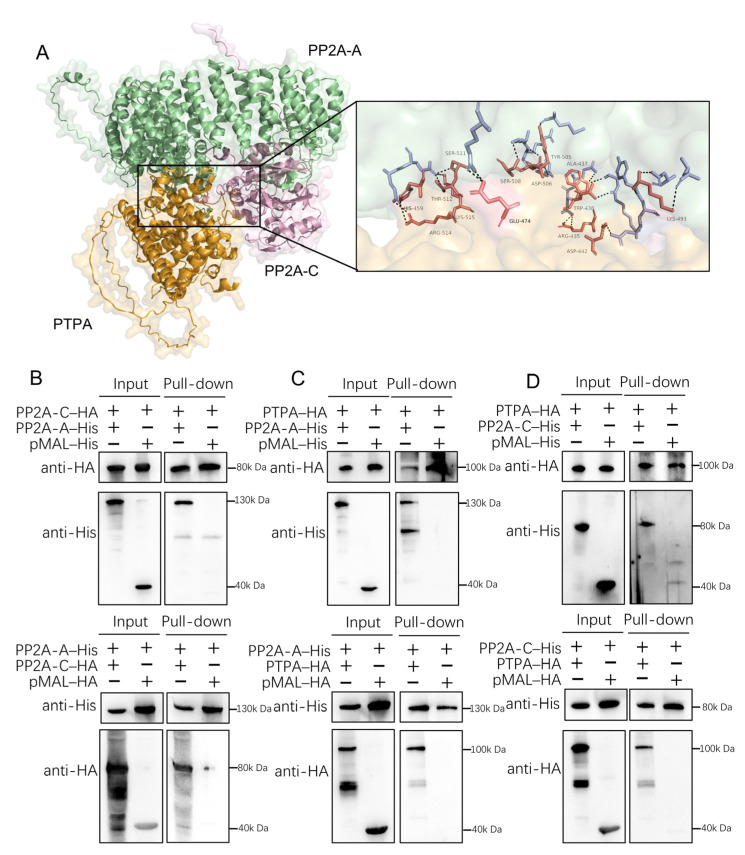
PTPA interacts with PP2A A and C subunits in *Toxoplasma gondii*. (**A**) Predicted interaction interface of the PTPA–PP2A A/C ternary complex modeled by AlphaFold3 (ipTM = 0.67, pTM = 0.69). PTPA (orange), PP2A A subunit (green), and PP2A C subunit (pink) exhibit extensive interfacial contacts. (**B**) Pull-down of PP2A A and C subunits. Anti-His magnetic beads incubated with PP2A-A–His (130 kDa) pulled down PP2A-C–HA, while anti-HA beads incubated with PP2A-C–HA (80 kDa) pulled down PP2A-A–His. Western blot (WB) confirmed specific interactions independent of the pMAL fusion tag (40 kDa). +, add; −, not add. (**C**) Interaction between PP2A A subunit and PTPA. WB analysis revealed binding of pMAL–PTPA–HA (~100 kDa) to PP2A-A–His. (**D**) Interaction between PP2A C subunit and PTPA. Pull-down assays demonstrated binding of pMAL–PTPA–HA to PP2A-C–HA.

Previous studies in eukaryotes have established that PTPA binds to the C subunit of protein phosphatase 2A (PP2A) to restore its phosphotyrosine phosphatase activity. Physical interaction between PTPA and PP2A has also been reported in mammalian cells and *Plasmodium*. To determine whether this interaction is conserved in *T. gondii*, we investigated the potential association of PTPA with the A and C subunits of PP2A. Using AlphaFold, we generated structural models of PTPA in complex with the PP2A A and C subunits (Figure 2A). The predicted model (ipTM = 0.67, pTM = 0.69) revealed distinct interfacial contacts between PTPA (orange) and both the PP2A A (green) and C (pink) subunits, supporting the likelihood of a tripartite interaction.

To experimentally validate these predictions, we engineered recombinant constructs on the pMAL plasmid backbone to express fusion proteins: pMAL–PTPA–HA (553 amino aa, ~100 kDa including the ~40 kDa pMAL tag), pMAL–PP2AA–His (766 aa, ~130 kDa), pMAL–PP2AC–His/HA (347 aa, ~80 kDa). SDS–PAGE analysis confirmed the purity of expressed proteins, with Coomassie-stained single bands matching expected molecular weights. Pairwise pull-down assays were conducted to test interactions between (i) PP2A A and C subunits (Figure 2B), (ii) a PP2A A subunit and PTPA (Figure 2C), and (iii) a PP2A C subunit and PTPA (Figure 2D). Notably, all interactions were specific to the target proteins and independent of the pMAL backbone. These findings align with the AlphaFold predictions, providing convergent structural and biochemical evidence for a conserved PTPA–PP2A interaction network in *T. gondii*.

### 3.3. Conditional Depletion of PTPA Protein Impairs the Lytic Cycle of Toxoplasma gondii

**Figure 3:**
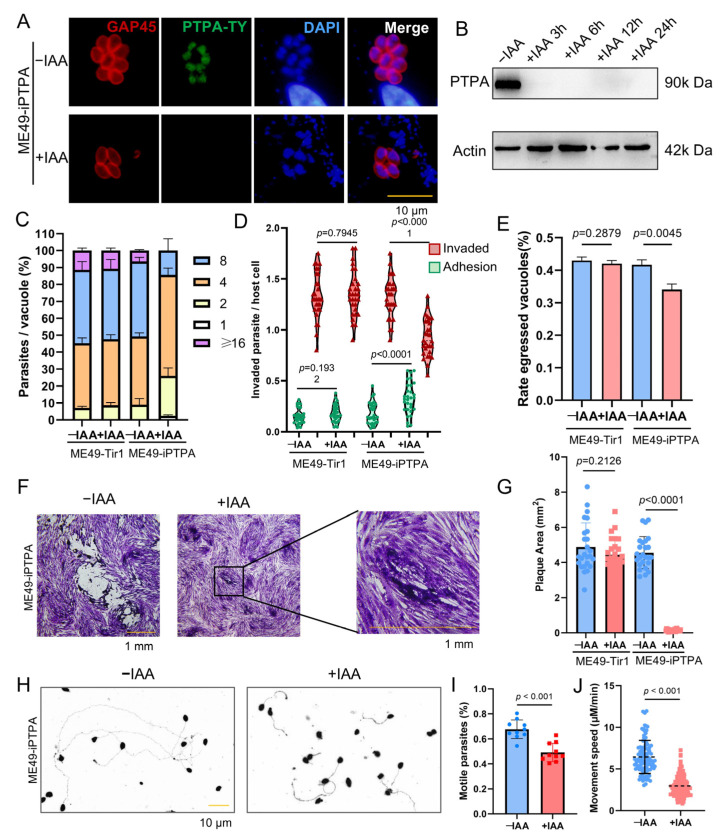
PTPA is essential for *Toxoplasma gondii* lytic cycle progression. (**A**) ME49-iPTPA was treated with 500 μM IAA or left untreated. IFA with anti-Ty (green) and anti-GAP45 (red) antibodies confirmed nuclear localization of PTPA. Scale bars: 10 μm. (**B**) WB analysis verified efficient degradation of PTPA (~90 kDa) within 3 h post-IAA treatment. (**C**) Proliferation assays showed significant growth inhibition in PTPA-depleted parasites after 24 h IAA treatment compared with untreated parasites. Means ± SEM of three independent experiments, each with three replicates. A total of 100 PVs were analyzed for each replicate in each experiment. Means ± SD of three independent experiments, each with three replicates, unpaired two-tailed Student’s *t*-test. (**D**) Invasion assays (SAG1/GAP45 staining) demonstrated reduced invasion and increased adhesion following 12 h IAA treatment. Means ± SD of three independent experiments, each with three replicates, unpaired two-tailed Student’s *t*-test. (**E**) Egress efficiency was severely impaired in PTPA-deficient parasites after 12 h IAA treatment compared with untreated parasites. Means ± SD of three independent experiments, each with three replicates, unpaired two-tailed Student’s *t*-test. (**F**,**G**) Plaque assays revealed attenuated plaque size after 10-day IAA treatment compared with untreated parasites. Means ± SD of three independent experiments, each with three replicates, unpaired two-tailed Student’s *t*-test. Scale bars: 1 mm. (**H**–**J**) Gliding motility assays (including poly-l-lysine-coated slides and 5% ethanol stimulation), quantified using anti-SAG1 staining, showed decreased gliding frequency and speed in PTPA-depleted parasites after 12 h IAA treatment compared with untreated parasites. Means ± SD of three independent experiments, each with three replicates, unpaired two-tailed Student’s *t*-test. Scale bars: 10 μm.

In previous studies, we reported that deletion of the PTPA protein in the RH strain of *T. gondii* disrupts iron depletion-induced bradyzoite differentiation. Given the critical role of PTPA in bradyzoite development, we further investigated its functional significance in the type II ME49 strain. Using the ME49*Δku80::TIR1* strain, the auxin-inducible degradation (AID) system was employed to conditionally deplete PTPA by tagging its C-terminus with a Ty-AID epitope (designated ME49-iPTPA). Immunofluorescence assay (IFA) confirmed nuclear localization of PTPA (Figure 3A). Upon addition of indole-3-acetic acid (IAA) to the culture medium, nuclear PTPA signals were undetectable by IFA, and western blot (WB) analysis demonstrated efficient degradation of PTPA within three hours (Figure 3B).

Functional characterization of PTPA-depleted parasites revealed profound impacts on key lytic cycle processes. Depletion of PTPA significantly impaired parasite proliferation (Figure 3C) and plaque-forming capacity compared to the parental strain (Figure 3F,G). Furthermore, PTPA-deficient parasites exhibited reduced host cell invasion efficiency, enhanced adhesion capability (Figure 3D), and diminished egress capacity (Figure 3E). Given the dependence of invasion and egress on parasite motility, we assessed gliding motility and observed a marked reduction in both the frequency of gliding trails and gliding speed in PTPA-depleted parasites (Figure 3H–J).

**Figure 4:**
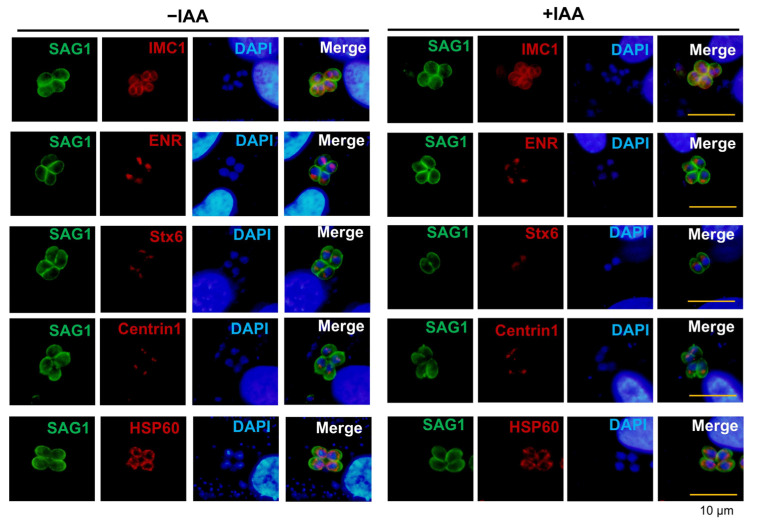
PTPA depletion does not disrupt organellar integrity in *Toxoplasma gondii*. IFA analysis of ME49-iPTPA parasites ± IAA treatment reveals no morphological changes (red) in the inner membrane complex (IMC1), apicoplast (ENR), Golgi apparatus (Stx6), centrosome (Centrin1), or mitochondrion (HSP60). The plasma membrane was stained with anti-SAG1 antibodies (green). Scale bars: 10 μm.

Intriguingly, despite the broad disruption of lytic cycle progression caused by PTPA depletion, critical cellular structures remained unaffected. Immunostaining of major organelles—including the inner membrane complex (IMC1), centrosome (Centrin1), apicoplast (ENR), mitochondrion (HSP60), and Golgi apparatus (Stx6)—revealed no defects in daughter cell formation, apicoplast morphology, or mitochondrial and Golgi architecture (Figure 4). These findings highlight the specific regulatory role of PTPA in parasite motility and lytic cycle, distinct from its dispensability in organelle biogenesis and structural maintenance.

### 3.4. PTPA Depletion Impairs Tachyzoite-to-Bradyzoite Conversion in Toxoplasma gondii

**Figure 5:**
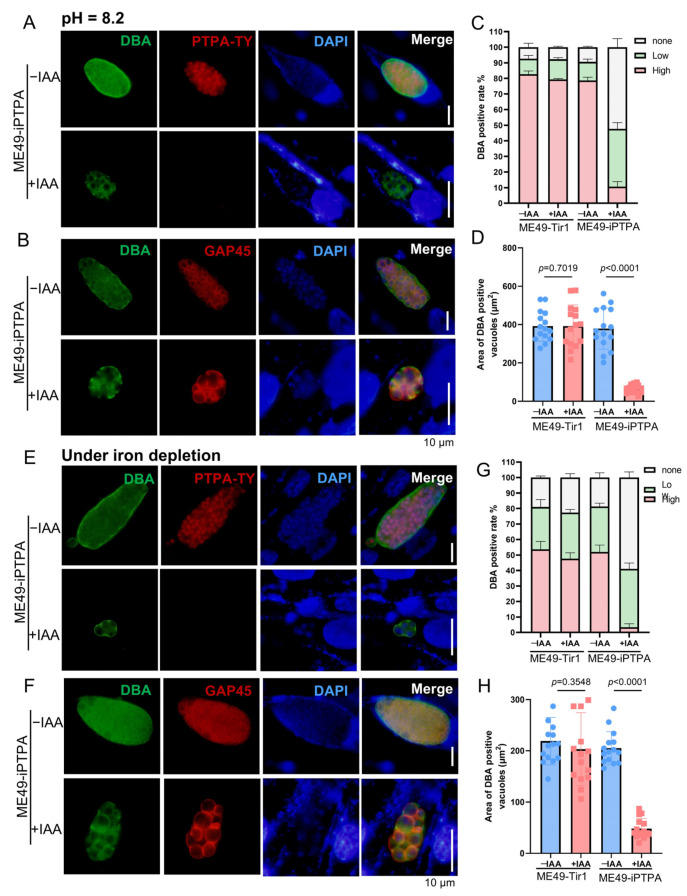
PTPA depletion impairs tachyzoite-to-bradyzoite differentiation in *Toxoplasma gondii*. (**A**,**B**) ME49-iPTPA parasites cultured in alkaline medium (pH 8.2) ± IAA for 3 days were subjected to IFA using DBA (green) to label cyst walls and anti-Ty (red, (**A**)) or anti-GAP45 (red, (**B**)) antibodies to detect PTPA or plasma membranes, respectively. Scale bars: 10 μm. (**C**,**D**) Quantification of DBA-positive PVs under alkaline stress. PTPA depletion significantly reduced both the proportion of DBA-positive PVs (**C**) and their cross-sectional area (**D**), indicating defective bradyzoite differentiation. Data represent means ± SEM (*p* < 0.01, Student’s *t*-test). Means ± SD of three independent experiments, each with three replicates, unpaired two-tailed Student’s *t*-test. (**E**,**F**) ME49-iPTPA parasites cultured under iron depletion (DFO-supplemented) ± IAA for 3 days were analyzed by IFA using DBA (green) and anti-Ty (red, (**E**)) or anti-GAP45 (red, (**F**)) antibodies. Scale bars: 10 μm. (**G**,**H**) Quantification of DBA-positive PVs under iron-depletion stress. PTPA depletion markedly decreased the proportion (**G**) and size (**H**) of DBA-positive PVs. Data represent means ± SEM (*p* < 0.001, Student’s *t*-test). Means ± SD of three independent experiments, each with three replicates, unpaired two-tailed Student’s *t*-test.

Previous work demonstrated that iron depletion induces spontaneous bradyzoite differentiation in the RH strain of *T. gondii*, a process disrupted by PTPA deletion. Here, we extend these findings to the type II ME49 strain, revealing that PTPA is indispensable for stress-induced tachyzoite-to-bradyzoite conversion across genotype. To assess PTPA’s role, ME49-iPTPA parasites were subjected to bradyzoite-inducing conditions: alkaline stress (pH 8.2) or iron depletion (via deferoxamine mesylate, DFO). Upon IAA-mediated PTPA degradation, Dolichos biflorus agglutinin (DBA) staining revealed a significant reduction in strongly DBA-positive parasitophorous vacuoles (PVs), indicative of mature bradyzoites, alongside a marked increase in weakly DBA-positive PVs (Figure 5A,C,E,G). These data establish that PTPA depletion blocks bradyzoite differentiation despite pro-differentiation stimuli. Notably, PVs retaining DBA positivity in PTPA-depleted parasites exhibited reduced dimensions (Figure 5D,H), suggesting impaired vacuolar maturation. Furthermore, immunostaining of the plasma membrane marker GAP45 exposed striking morphological anomalies: parasites lost their characteristic tapered shape and displayed aberrant swelling under stress conditions (Figure 5B,F). Importantly, this phenotype was absent in PTPA-depleted parasites under normal culture, highlighting a context-specific role for PTPA in stress adaptation.

### 3.5. PTPA Depletion Triggers Metabolic Dysregulation and Ultrastructural Disintegration in Toxoplasma gondii

**Figure 6:**
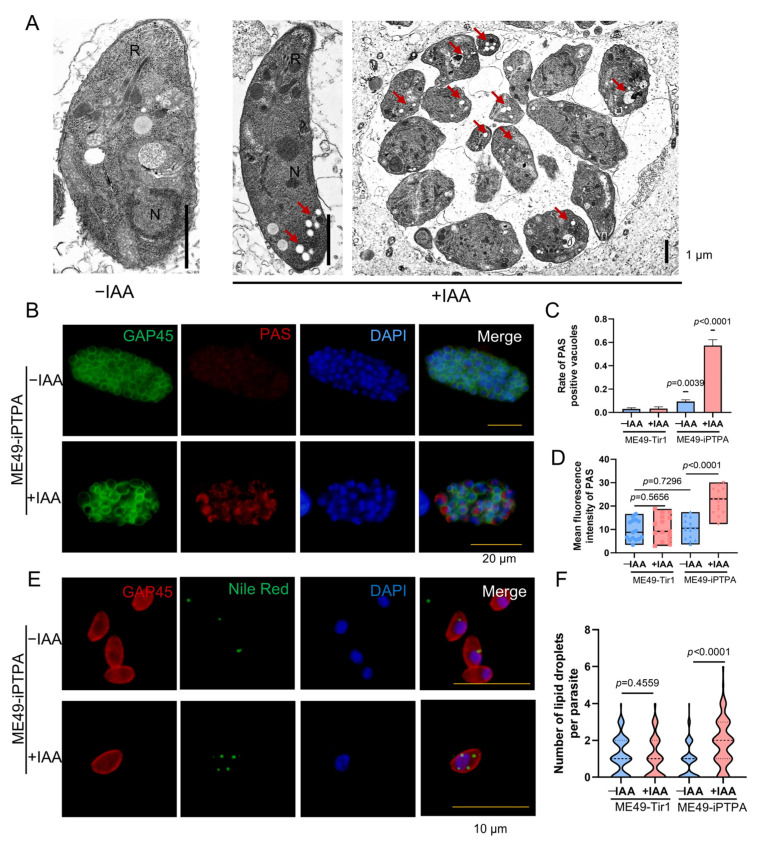
PTPA depletion triggers amylopectin and lipid droplet accumulation in *Toxoplasma gondii* tachyzoites. (**A**) Transmission electron microscopy (TEM) of ME49-iPTPA tachyzoites ± IAA treatment. R: rhoptries; N: nucleus. Scale bars: 1 μm. (**B**–**D**) PAS staining revealed increased amylopectin granules in ME49-iPTPA tachyzoites subjected to 3-day IAA treatment, compared with untreated parasites. Parasites were labeled with anti-GAP45 (green, plasma membrane) and PAS (red, amylopectin). PTPA depletion (+IAA) induced punctate PAS signals in PVs, with significantly increased PAS-positive PVs (**C**) and fluorescence intensity (**D**) (means ± SEM; Student’s *t*-test). Scale bars: 20 μm. (**E**,**F**) Compared with untreated controls, ME49-iΔPTPA tachyzoites subjected to 24 h IAA treatment exhibited significantly increased lipid droplets count per parasite, as quantified by Nile Red staining. Parasites were labeled with anti-GAP45 (red) and Nile Red (green). Means ± SEM; Student’s *t*-test. Scale bars: 10 μm.

**Figure 7:**
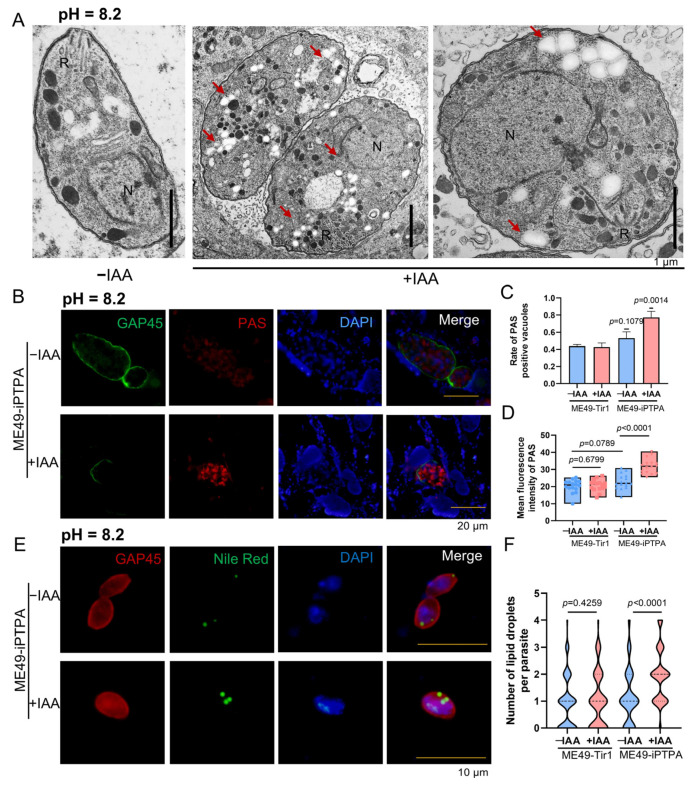
PTPA depletion induces amylopectin and lipid droplet accumulation in *Toxoplasma gondii* bradyzoites. (**A**) TEM of ME49-iPTPA bradyzoites (±IAA treatment) differentiated in pH 8.2 medium for 3 days. R: rhoptries; N: nucleus. Scale bars: 1 μm. (**B**–**D**) PAS staining of amylopectin granules in ME49-iPTPA bradyzoites. Parasites were labeled with anti-GAP45 (green, plasma membrane) and PAS (red, amylopectin). PTPA depletion (+IAA) resulted in punctate PAS signals within PVs, with significantly elevated PAS-positive PV frequency (**C**) and fluorescence intensity (**D**) compared with untreated (−IAA) parasites (means ± SEM; Student’s *t*-test). Scale bars: 20 μm. (**E**,**F**) Nile Red staining of lipid droplets in ME49-iPTPA bradyzoites ± IAA treatment. Parasites were labeled with anti-GAP45 (red) and Nile Red (green). PTPA depletion (+IAA) triggered an increase in lipid droplet abundance per parasite (**F**) compared with untreated (−IAA) parasites (means ± SEM; Student’s *t*-test). Scale bars: 10 μm.

Depletion of PTPA in *T. gondii*, while preserving major organelle architecture in tachyzoites, induces profound metabolic dysregulation and ultrastructural anomalies in bradyzoites. Transmission electron microscopy (TEM) of IAA-treated ME49-iPTPA parasites revealed abundant electron-lucent vacuoles (Figure 6A), hypothesized to represent amylopectin granules or lipid droplets. Periodic acid–Schiff (PAS) staining confirmed amylopectin accumulation in PVs of PTPA-depleted tachyzoites (Figure 6B), with punctate PAS signals and significantly higher fluorescence intensity compared to untreated controls (Figure 6C,D). Concurrently, Nile Red staining demonstrated a striking increase in lipid droplet numbers in IAA-treated tachyzoites (4–5 droplets/parasite vs. ≤1 in controls, Figure 6E,F). In bradyzoites differentiated under alkaline stress (pH 8.2), PTPA depletion exacerbated these phenotypes: TEM analysis revealed a marked proliferation of electron-lucent vacuoles alongside severe cellular swelling and organelle disorganization (Figure 7A). PAS and Nile Red staining mirrored tachyzoite trends, with elevated amylopectin storage (Figure 7B–D) and lipid droplet abundance (Figure 7E,F) in PTPA-deficient bradyzoites. Although *T. gondii* stores starch granules in the cytoplasm upon differentiation into bradyzoites, observable as hypodense regions under TEM, PTPA-deficient parasites exhibited significantly more hypodense granules compared to wild-type controls. Notably, the structural disintegration observed in stressed bradyzoites—absent in unstressed PTPA-depleted parasites—highlights a stage-specific reliance on PTPA for maintaining metabolic homeostasis and cellular integrity during stress adaptation. These findings collectively position PTPA as a critical regulator of energy storage dynamics and structural stability across *T. gondii* life stages.

### 3.6. Stage-Specific Proteomic Reveal PTPA’s Role in Toxoplasma gondii Survival and Development

**Figure 8:**
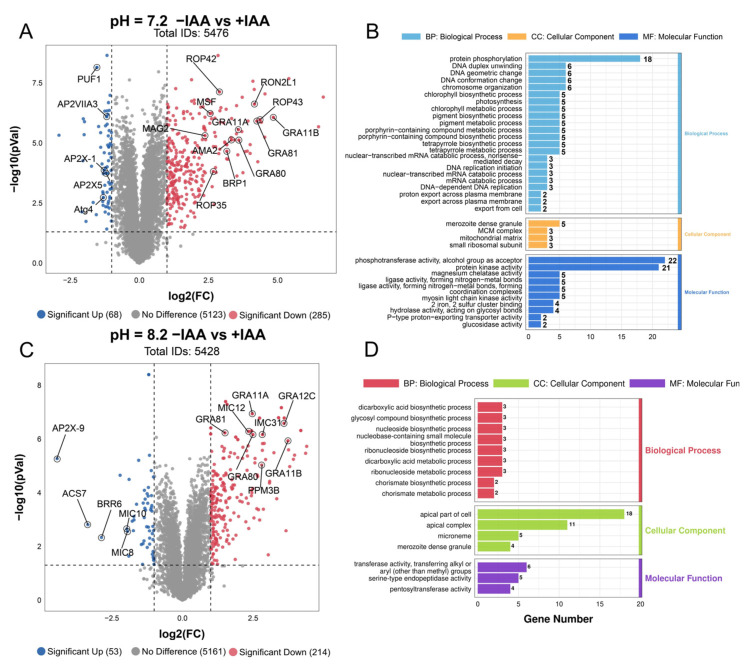
Proteomic profiling reveals stage-specific functional impacts of PTPA depletion in *Toxoplasma gondii*. (**A**) Volcano plot of tachyzoite proteomes under normal culture conditions comparing PTPA-depleted (+IAA) and untreated (−IAA) ME49-iPTPA parasites. Proteins with |log2(fold change)| ≥ 1 and *p* < 0.05 (red: upregulated; blue: downregulated). (**B**) Gene ontology (GO) enrichment analysis of differentially expressed proteins in tachyzoites. (**C**) Volcano plot of bradyzoite proteomes under alkaline stress (pH 8.2) comparing PTPA-depleted (+IAA) and untreated (−IAA) ME49-iPTPA parasites. (**D**) GO enrichment analysis of bradyzoite proteomes under alkaline stress.

To comprehensively assess the consequences of PTPA depletion on *T. gondii*, we conducted comparative proteomic profiling of ME49-iPTPA tachyzoites and alkaline-stress-induced bradyzoites under IAA-mediated protein degradation. In tachyzoites cultured under standard conditions, proteomic analysis identified 5476 proteins (Figure 8A), with 285 significantly upregulated (log2FC ≥ 1, *p* <0.05) and 68 downregulated (log2FC ≤ −1). Notably, upregulated proteins included bradyzoite-specific markers such as AMA2 and BRP1, as well as merozoite-stage protein like GRA11B, GRA80, and GRA81, suggesting aberrant developmental activation. Downregulated proteins were enriched in AP2 transcription factors (AP2X5, AP2X-1, and AP2VIIA3), implicating transcriptional dysregulation. Gene ontology (GO) analysis further revealed that PTPA depletion disrupted molecular functions critical for parasite, including phosphotransferase activity and protein kinase activity. Biological processes like protein phosphorylation were markedly perturbed, while cellular components such as merozoite dense granule, mitochondrial matrix, and MCM complex were significantly impaired (Figure 8B).

In alkaline-stress-adapted bradyzoites, proteomic analysis detected 5528 proteins, with 214 upregulated and 53 downregulated (Figure 8C). Similar to tachyzoites, merozoite-specific GRA genes (GRA11B, GRA80, and GRA81) were upregulated, alongside suppression of the transcriptional regulator AP2X-9. GO enrichment revealed stage-specific functional divergences upon PTPA depletion (Figure 8D). In bradyzoites, cellular components such as the apical part of cell, apical complex, and micronemes were severely compromised. This contrasted sharply with tachyzoites, where apical annuli perturbations were absent. Molecular functions in bradyzoites, including alkyl/aryl transferase and serine-type endopeptidase activity, were broadly impaired, while stress-adaptive biological processes like dicarboxylic acid biosynthesis, chorismate metabolism, and nucleotide biosynthesis collapsed. Notably, phosphorylation-related pathways, while extensively disrupted in tachyzoites (include phosphotransferases and protein kinases), remained largely intact in bradyzoites, underscoring a life stage-dependent partition of PTPA’s regulatory roles. Conversely, the unique vulnerability of apical components in bradyzoites highlights their stage-specific reliance on PTPA for maintaining secretory competence during chronic infection.

PTPA depletion drives widespread proteomic remodeling in *T. gondii*, with tachyzoites displaying phosphorylation disruptions and bradyzoites experiencing metabolic breakdown. Persistent upregulation of merozoite-specific GRA genes across life stages implies a conserved stress-survival strategy, while suppression of AP2 transcription factors underscores PTPA’s pivotal role in developmental regulation.

### 3.7. LB-100 Targets PTPA–PP2A Axis to Block Toxoplasma gondii

**Figure 9:**
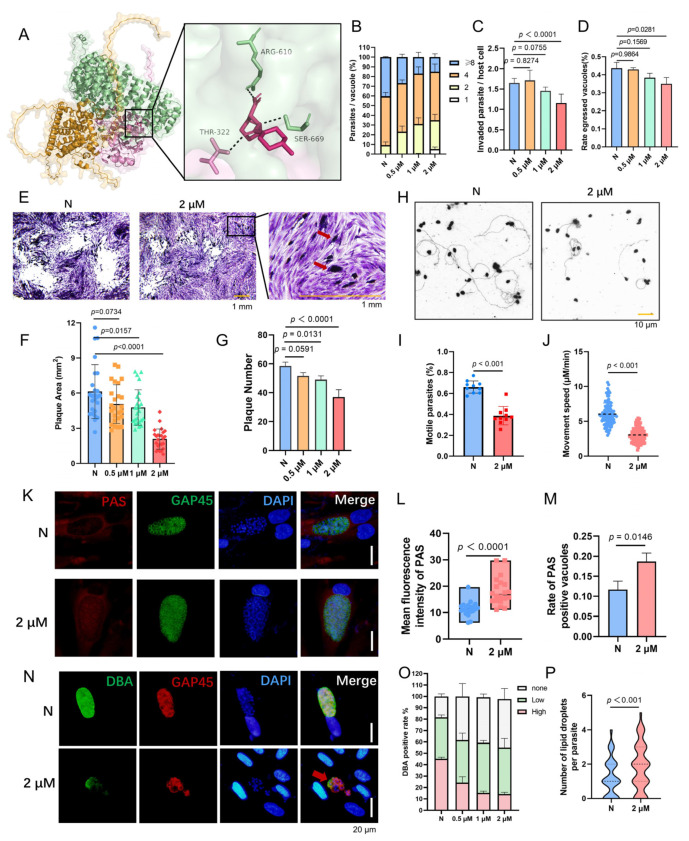
LB-100 inhibits *Toxoplasma gondii* proliferation, differentiation, and metabolic homeostasis. (**A**) Molecular docking of LB-100 to the PTPA–PP2A A/C ternary complex using CB-Dock2 revealed strong binding (binding energy: −7.2 kcal/mol). (**B**) Dose-dependent suppression of tachyzoite proliferation by LB-100 (24 h). Means ± SD of three independent experiments, each with three replicates, unpaired two-tailed Student’s *t*-test. (**C**,**D**) LB-100 (2 μM) significantly impaired parasite invasion (**C**) and egress (**D**) after 12 h treatment. Means ± SD of three independent experiments, each with three replicates, unpaired two-tailed Student’s *t*-test. (**E**–**G**) Plaque assays demonstrated reduced plaque size (**F**) and number (**G**) after 12-day LB-100 (2 μM) treatment. Trapped PVs indicated failed egress (**E**). Means ± SEM; Student’s *t*-test. Scale bars: 1 mm. (**H**–**J**) Gliding motility assays (15 min stimulation with 5% ethanol) showed LB-100 (2 μM) reduced gliding frequency (**I**) and speed (**J**). Trails visualized via SAG1 antibody staining (**H**). Means ± SEM; Student’s *t*-test. Scale bars: 10 μm. (**K**–**M**) PAS staining of amylopectin granules in LB-100-treated tachyzoites (2 μM, 2-day). Anti-GAP45 (green) and PAS (red) revealed amylopectin accumulation in PVs (**K**), with elevated PAS-positive PVs (**L**) and fluorescence intensity (**M**). Means ± SEM; Student’s *t*-test. Scale bars: 20 μm. (**N**,**O**) Alkaline stress-induced bradyzoites (pH 8.2, 3-day) treated with LB-100 (2 μM) showed reduced cyst wall formation via DBA (green) and anti-GAP45 (red) staining (**N**). Quantification confirmed fewer DBA-positive PVs. Means ± SEM; Student’s *t*-test. Scale bars: 20 μm. (**P**) Nile Red staining of lipid droplets in LB-100-treated tachyzoites (2 μM, 24 h) revealed an increase in lipid droplet abundance. Means ± SEM; Student’s *t*-test.

The PTPA protein, essential for *T. gondii* pathogenesis and a promising yet unexplored drug target, is hypothesized to exert its critical functions through PP2A activation. While no direct PTPA inhibitors exist, we investigated LB-100, a water-soluble, clinically tested PP2A inhibitor with established antitumor activity, as a candidate to disrupt the PTPA–PP2A axis. Molecular docking revealed strong binding of LB-100 to the PTPA–PP2A complex (binding energy: −7.2 kcal/mol), mediated by hydrogen bonds with Arg610 and Ser669 in the PP2A A subunit and Thr322 in the PP2A C subunit (Figure 9A). In vitro, LB-100 exhibited dose-dependent inhibition of tachyzoite proliferation (Figure 9B), with 2 μM treatment significantly impairing invasion (Figure 9C), egress (Figure 9D), and gliding motility (Figure 9H–J). Plaque assays demonstrated reduced plaque size and number, accompanied by trapped PVs indicative of failed egress (Figure 9E–G). Notably, LB-100 treatment phenocopied PTPA depletion across multiple metrics: PAS staining revealed amylopectin granule accumulation in PVs (Figure 9K–M), while Nile Red assays identified lipid droplet proliferation (Figure 9P). Furthermore, alkaline stress-induced bradyzoite differentiation was markedly suppressed by LB-100, with immunofluorescence microscopy revealing vacuolar swelling and parasite deformation akin to PTPA-deficient strains (Figure 9N,O).

These findings demonstrate that LB-100 phenocopies PTPA depletion by disrupting PP2A activity, thereby crippling both acute lytic cycle progression and chronic stage adaptation. These findings confirm again the PTPA–PP2A interaction as critical for parasite survival. These findings validate the PTPA–PP2A axis as a high-potential therapeutic target, with LB-100 serving as an inhibitor.

## 4. Discussion

In this study, we elucidate the indispensable role of *Toxoplasma gondii* phosphotyrosyl phosphatase activator (TgPTPA) in parasite biology. Through conditional gene depletion and proteomic approaches, we demonstrate that TgPTPA orchestrates critical processes across the parasite’s lytic cycle and stress-responsive differentiation. Functional characterization reveals that TgPTPA forms a stable ternary complex with both PP2A A and C subunits, ensuring dephosphorylation signaling fidelity required for tachyzoite proliferation, invasion, egress, and gliding motility. Remarkably, TgPTPA is essential for bradyzoite maturation under stressors such as iron depletion or alkaline pH, where its absence abrogates cyst wall formation and disrupts metabolic adaptation. These findings position TgPTPA as a linchpin of *T. gondii* pathogenesis, bridging post-translational regulation to lifecycle plasticity, and unveil novel therapeutic strategies targeting its stage-specific dependencies.

The phosphotyrosyl phosphatase activator (PTPA) retains a highly conserved core domain across eukaryotes, yet functional divergence among species likely arises from disordered regions flanking this structural motif. Phylogenetically, TgPTPA clusters with red algal homologs, suggesting adaptations to apicomplexan-specific metabolic demands, such as starch/lipid metabolism and stress response regulation. Size variations in TgPTPA homologs across *T. gondii* genotypes (I, II, and III) correlate with phenotypic diversity, potentially influencing virulence, replication rates, and host adaptation.

The functional conservation of PTPA in *T. gondii* is exemplified by its canonical role in activating and interacting with protein phosphatase 2A (PP2A), a mechanism widely documented across eukaryotes—including humans [15], *Arabidopsis* [25], yeast [26], and *Plasmodium* [16]. In *T. gondii*, this evolutionary preservation is further validated by the direct interaction between TgPTPA and both PP2A A and C subunits, as demonstrated through structural modeling and reciprocal pull-down assays. Beyond PP2A, proteomic profiling of PTPA-depleted tachyzoites revealed widespread dysregulation of phosphorylation-associated pathways, suggesting that TgPTPA may coordinate additional phosphatases or phosphorylation-dependent networks. Notably, the nuclear localization of TgPTPA contrasts with the cytoplasmic distribution of the PP2A holoenzyme [18], raising questions about the spatial dynamics of their interaction. This apparent compartmentalization implies a nucleocytoplasmic shuttling mechanism, where either PP2A transiently enters the nucleus or TgPTPA exports to the cytoplasm to facilitate complex assembly—a regulatory paradigm requiring further interrogation. Such spatial orchestration may fine-tune phosphatase activity in response to developmental or environmental cues, underscoring the adaptability of this conserved signaling axis in apicomplexan parasites.

**Figure 10:**
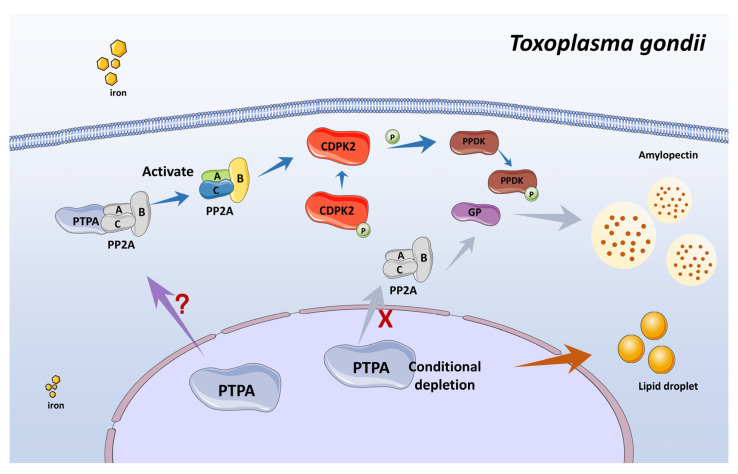
Schematic of the PTPA-activated PP2A network regulating amylopectin metabolism in *Toxoplasma gondii.* The PTPA protein activates PP2A phosphatase activity, prompting PP2A to dephosphorylate downstream substrates—including CDPK2. CDPK2 mediates phosphorylation of enzymes involved in amylopectin synthesis, such as GP (glycogen phosphorylase) and PPDK (pyruvate phosphate dikinase). Upon conditional depletion of PTPA, reduced PP2A activity impairs the function of these substrates, leading to pathological accumulation of amylopectin granules. Excessive amylopectin accumulation compromises parasite fitness by obstructing the tachyzoite-to-bradyzoite conversion. Concurrently, PTPA depletion elevates lipid droplet abundance via undefined mechanisms. Furthermore, PTPA expression levels appear to correlate with intracellular iron homeostasis in the parasite.

The functional specificity of TgPTPA is underscored by its regulatory roles in amylopectin and lipid metabolism, as well as its critical influence on stress-mediated bradyzoite differentiation. A hallmark of bradyzoites is their ability to accumulate amylopectin granules in the cytosol [3], which serve as energy reservoirs to sustain parasite viability under nutrient-limited conditions [27]. PTPA depletion triggers aberrant amylopectin accumulation in both tachyzoites and bradyzoites, likely due to disrupted PP2A activation. PP2A regulates amylopectin metabolism via dephosphorylation of calcium-dependent protein kinase 2 (CDPK2) at Ser679 [18]. Loss of CDPK2 results in starch hyperaccumulation and death of chronic-stage parasites [28]. Phosphorylation of several starch-metabolic enzymes relies on CDPK2 activity [28]. Intriguingly, PTPA depletion also induces lipid droplet proliferation across lifecycle stages. While Renaud et al. [29] observed iron depletion mediated lipid biosynthesis in *T. gondii*, our data suggest that PTPA loss can also lead to this phenomenon (Figure 10). Furthermore, proteomic profiling detected ectopic expression of merozoite-stage proteins in PTPA-depleted tachyzoites and bradyzoites, suggesting disrupted stage transition fidelity. We hypothesize that TgPTPA ensures orderly lifecycle progression by modulating dephosphorylation-dependent signaling cascades. Stressors typically drive tachyzoite-to-bradyzoite conversion or bradyzoite-to-merozoite conversion [30,31]; however, PTPA deficiency destabilizes this stepwise regulation, leading to developmental arrest. The pronounced structural and metabolic anomalies in PTPA-deficient bradyzoites, including vacuolar swelling and organelle disarray, further highlight its pivotal role in stress-responsive adaptation.

While targeted inhibitors for TgPTPA remain elusive, the PP2A inhibitor LB-100 demonstrates potent anti-Toxoplasma activity. Derived from norcantharidin, a synthetic analog of the traditional Chinese medicine compound cantharidin [32], LB-100 is a water-soluble [33], competitive PP2A inhibitor that binds directly to the catalytic subunit (PP2A-C), suppressing its phosphatase activity [34]. At 2 μM, LB-100 phenocopies PTPA depletion, impairing parasite proliferation, invasion, and stress-induced differentiation, thereby confirming that PP2A activation constitutes the predominant function of TgPTPA. Zhao et al. reported that PP2A inhibition by okadaic acid triggers polysaccharide accumulation in tachyzoites, and the loss of the catalytic subunit α (*PP2Acα*) and the regulatory B subunit (*B′/PR61*) of PP2A also led to the accumulation of polysaccharide granules in the tachyzoites [35]. This implicates PP2A substrate dephosphorylation (e.g., CDPK2) in regulating energy storage and stage transitions. The convergence of PTPA depletion and LB-100-mediated PP2A inhibition underscores the PTPA–PP2A axis’s therapeutic promise.

Collectively, TgPTPA emerges as a linchpin integrating metabolic regulation, phosphorylation signaling, and stage-specific differentiation, offering dual therapeutic targets to disrupt both acute and chronic toxoplasmosis.

## Data Availability

The original contributions presented in this study are included in the article/Supplementary Materials. Further inquiries can be directed to the corresponding author.

## Supplementary Materials

The following supporting information can be downloaded at: https://www.mdpi.com/article/10.3390/cells14110835/s1, Table S1: The primers used in this study; Table S2: Proteomic data.
