# Supplementary material for: PTPA Governs Stress-Responsive Differentiation and Metabolic Homeostasis in Toxoplasma gondii
Source: Cells. 2025 Jun 3;14(11):835. doi: 10.3390/cells14110835 (PMC12155429; doi:10.3390/cells14110835)
Supplement: Supplementary file 1 [file cells-14-00835-s001.zip › Table S1 The primers used in this study.pdf]

Table S1: The primers used in this study

283720-AID-U6-R	CCCTACTTCCGGCTGTCTCCAACTTGACATCCCCATTTA
283720-AID-AMP-F	GGAGACAGCCGGAAGTAGGGGTTTTAGAGCTAGAAATAG
283720-42bp-F	TTCGGCAAGTACTTTCCTTTCTCGCGAAAGGCCGCCGACGTCCCTAGGTACCCGTACGAC
283720-42bp-R	GGAGGAGGTGAGCAGCAGCGACAGAGAGAGGAAAGCGAGGAAACTAGTGGATCGATCCCC
283720-JD-F	TGCTGTATGACATCTCGGGCG
283720-JD-R	GACCTCGCAGTGCTCATACG
pMAL-PP2AA-HIS-F	CCATGGGCGGCCGCGATATCATGGCGGAACATCTCTCCTT
pMAL-PP2AA-HIS-R	CCTGCAGGGAATTCGGATCCTGAGCGCGCCGCAGCGAGAG
pMAL-PP2AC-HIS-F	CCATGGGCGGCCGCGATATCATGTCGGGTGCAACTCCAGG
pMAL-PP2AC-HIS-R	CCTGCAGGGAATTCGGATCCCAGGAAGTAGTCCGGAATTC
pMAL-PTPA-HA-F	CCATGGGCGGCCGCGATATCATGCCCAGAGATCCGGAACT
pMAL-PTPA-HA-R	TCTGGAACATCGTAAGGATAGACGTCGGCGGCCTTTCGCG
pMAL-HA-R	ATATCGCGGCCGCCCATGG
pMAL-HA-F	TATCCTTACGATGTTCCAGA
pMAL-His-F	GATATCGCGGCCGCCCATGG
pMAL-His-R	GGATCCGAATTCCCTGCAGG
pMAL-JD-F	GGTCGTCAGACTGTCGATGAAGCC
pMAL-JD-R	TGTCCTACTCAGGAGAGCGTTCAC
